# Sialic Acids in the Immune Response during Sepsis

**DOI:** 10.3389/fimmu.2017.01601

**Published:** 2017-11-21

**Authors:** Yan-Cun Liu, Mu-Ming Yu, Yan-Fen Chai, Song-Tao Shou

**Affiliations:** ^1^Department of Emergency Medicine, Tianjin Medical University General Hospital, Tianjin, China

**Keywords:** sialic acid-binding immunoglobulin-type lectins, sepsis, infection, sialic acid, inflammation

## Abstract

Sialic acid-binding immunoglobulin-type lectins (Siglecs) are a group of cell surface transmembrane receptors expressed on immune cells, and regulate immune balance in inflammatory diseases. Sepsis is a life-threatened inflammatory syndrome induced by infection, and the pathogenesis of sepsis includes immune dysregulation, inflammation, and coagulation disorder. Here, we reviewed the various roles acted by Siglecs family in the pathogenesis of sepsis. Siglec-1, Siglec-5, and Siglec-14 play bidirectional roles through modulation of inflammation and immunity. Siglec-2 regulates the immune balance during infection by modulating B cell and T cell response. Siglec-9 helps endocytosis of toll-like receptor 4, regulates macrophages polarization, and inhibits the function of neutrophils during infection. Siglec-10 inhibits danger-associated molecular patterns induced inflammation, helps the initiation of antigen response by T cells, and decreases B-1a cell population to weaken inflammation. Regulating the Siglecs function in the different stages of sepsis holds great potential in the therapy of sepsis.

## Introduction

Sialic acid-binding immunoglobulin-type lectins (Siglecs), a broad range of cell surface transmembrane receptors that contain 2–17 extracellular Ig domains, are found on the surface of both innate and adaptive immune cells (1). Through recognition of their glycan ligands, they are involved in the regulation of immune balance in sepsis, autoimmune diseases, and cancer (2–5). Siglecs can be divided into two groups. Group 1 consists of sialoadhesin, CD22, Siglec-4, and Siglec-15, which are conserved across mammals. Group 2 consists of CD33-related Siglecs that vary from species to species, and humans express a much larger variety of CD33-related Siglecs than rodents due to the loss of Siglecs genes in rodents (6). The extracellular Ig domains includes an amino-terminal V-set domain which contains the sialic acid-binding site, while the cytoplasmic domains have immunoreceptor tyrosine-based inhibitory motifs (ITIMs) which plays a key role in modulating function of immune cell *via* tyrosine phosphatases recruitment, such as the SH2 domain-containing protein tyrosine phosphatases SHP-1 and SHP-2 (2).

Sepsis is defined as life-threatening organ dysfunction induced by an uncontrolled host response to invading pathogens, which kills as many as one in four similar to acute myocardial infarction, stroke, or multiple injury, and is the leading cause of mortality of patients in ICU worldwide and (7–13). Some patients die rapidly from septic shock accompanied multiple organs dysfunction caused by the cytokines storm, while some patients survive the initial phase of sepsis but die from the secondary infection caused by immunosuppression state in the late time of sepsis (14–16). Thus, it can be seen that the dysregulation of immune function by immune cells contribute to the high mortality of sepsis. As important receptors in immune cells, Siglecs are involved in the pathogenesis and therapy of sepsis. Here, we present the recent developments at our understanding of the roles of some sepsis-related Siglecs family members (Siglec-1, Siglec-2, Siglec-5, Siglec-7, Siglec-9, Siglec-10, and Siglec-14) in immune regulation, and we also summarize current efforts to develop therapeutics targeting Siglecs for the treatment of sepsis (Table 1).

**Table 1:** Sialic acid-binding immunoglobulin-type lectins (Siglecs) related researches in sepsis.

Siglecs	Research methods	Mechanisms underlying	Study type	Results	Reference
Siglec-1	Deletion of Siglec-1	Inflammation↑ vascular leakage↑	*Plasmodium* infected mice	MODS↑ Death↑	Gupta et al. (19)
	Deletion of Siglec-1	IFN-I production↓ PD-L1↓; CD8^+^ T cell exhaustion (↓)	Mice with LCMV infection	Immunopathology↑	Shaabani et al. (20)
	Siglec-1↑ by LPS-induced tolerant	TGF-(↑)	RAW264.7 macrophages	Innate immunity (endotoxin tolerance↑)	Wu et al. (21)
		Virus laden macrophages contacts to trans-infect B-1 cells and migrates into lymph nodes	MLV or HIV-1 infected mice	Spread of infection	Sewald et al. (22)

Siglec-2	Soluble CD22	Elevated in serum	Gram-negative bacterial septic patients	Correlated with severity of sepsis	Jiang et al. (26)
	Deletion of Siglec-2	Chemokine↑	WNV infected mice	Accelerated infection	Ma et al. (28)

Siglec-5 and Siglec-14	Human THP-1 cells, monocyte, neutrophils	Activated p38, MAPK, and Akt signaling pathways	GBS infection	Paired receptor to regulate immune response	Ali et al. (32)
	Human tissue, THP-1 cells	Bind to Hsp70	LPS stimulation	Paired receptor to regulate immune response	Fong et al. (35)

Siglec-7	Ba/F3 cells	Bind to SOCS3	Ba/F3 cells	Regulate cytokine-induced proliferation	Orr et al. (42)

Siglec-9	BMDMs, 293T cells, TLR4-HEK cells	MyD88-specific manner	LPS stimulation	Negative regulation of TLR4 responses	Boyd et al. (47)
	Siglec-E knockout mice	NF-κB and MAPK p38 signal pathway	Infected with *Escherichia coli*	Provide immune balance	Wu et al. (48)
	RAW264.7 macrophages	MAPK(MEK)/ERK pathways	IL-4 stimulation	Arg-1↑	Higuchi et al. (52)
	Deletion of Siglec-E	Akt activation	Aerosol of LPS	Neutrophil recruitment to lung↓; ROS↑	McMillan et al. (53)
	Human PBMC-derived macrophages	HS9-Fab03 bind to Siglec-9 antigen	LPS stimulation	Pro-inflammatory cytokines↓	Chu et al. (57)

Siglec-10	BMDMs, CHO cells, THP-1 cells	MyD88 and p38 MAPK signaling pathways	*Campylobacter jejuni* infection	Anti-inflammatory↑	Stephenson et al. (59)
	Deletion of Siglec-G	Bind with CD24 and DAMPs	AAP-induced liver injury in mice	Negative regulation of inflammation	Chen et al. (62)
	Deletion of Siglec-G	Binds to the BCR of B-1a cells	Siglec-G^−/−^ mice	Apoptosis↓	Jellusova et al. (68)

## Siglec-1

Siglec-1, also named sialoadhesin (CD169), a myeloid-cell receptor expressed on macrophages, recognizes viral membrane gangliosides and regulates the immune response of infection especially human immunodeficiency virus (HIV) infection (17, 18). On the one hand, Siglec-1 controls the severe immunopathology in infection. A recent study showed that the deletion of Siglec-1 in the plasmodium-infected mice increased the inflammation and vascular leakage, which increased the possibility of multiple organ dysfunction syndrome (19). Another recent study in the lymphocytic choriomeningitis virus infection, the interferon (IFN)-I production decreased, and mice exhibited severe immunopathology and died quickly after the deletion of Siglec-1 (20). Siglec-1 also promotes transforming growth factor-β (TGF-β) production in the *in vitro* macrophages, which suppresses the innate immunity and induces the endotoxin tolerance (21). On the other hand, Siglec-1 also promotes spread of infection and helps virus escape from neutralization. A recent study from murine leukemia virus or HIV-1-infected mice indicated that, after the capture of viruses by Siglec-1 on macrophages, the virus laden macrophages contacted to trans-infect B-1 cells, which subsequently migrated into the lymph node and contributed to the spread of infection (22). In an *in vitro* study, HIV-1 particles were inadequately accessed by anti-gp120 broadly neutralizing antibodies and thus were less susceptible to neutralization in deep virus-containing compartments in the help of Siglec-1 (23). It can been seen that Siglec-1 controls the severe immunopathology through increasing the production of IFN-I and TGF-β, on the other side, it also promotes spread of virus infection at the same time. Therefore, Siglec-1 plays a bidirectional role in infection and acts as a potential target in the treatment of sepsis.

## Siglec-2

Siglec-2 (CD22) is a cell surface receptor expressed mostly on B cells, and regulates B cells proliferation, survival, signaling, and antibody production (24). A previous study using Siglec-2^−/−^ mice confirmed that the absence of Siglec-2 did not interfere with the severity of arthritis, survival, bacterial clearance, and the inflammatory response during *Staphylococcus aureus* infection (25). However, with the gradual progress of Siglecs research in sepsis, it seems that Siglec-2 is closely associated with the development of sepsis. First, serum soluble CD22, a fragment of Siglec-2, was significantly elevated in patients with gram-negative bacterial sepsis and was correlated with the severity of sepsis (26). Second, in septic patients, miR-19a in B cells was up-regulated, and it comprised a feedback loop with Siglec-2 for B cell response. That provided a potential therapeutic target to restore the immune homeostasis in sepsis (27). What is more, a recent Siglec-2^−/−^ mice study confirmed that Siglec-2 helped to control West Nile virus infection through CD8 T cells response, promoted lymphocyte migration into the draining lymph nodes, and affected chemotaxis *via* controlling chemokine production (28). Siglec-2 specific immunotoxins have been used in clinical studies for hairy cell leukemia and autoimmune diseases (29, 30), however, studies on the sepsis is still lacking. To sum up, Siglec-2 is involved in the immune balance of sepsis through regulating B cell response and controlling chemokine production, and Siglec-2 targeting therapy holds a great potential for the treatment of sepsis.

## Siglec-5 and Siglec-14

**Figure 1:**
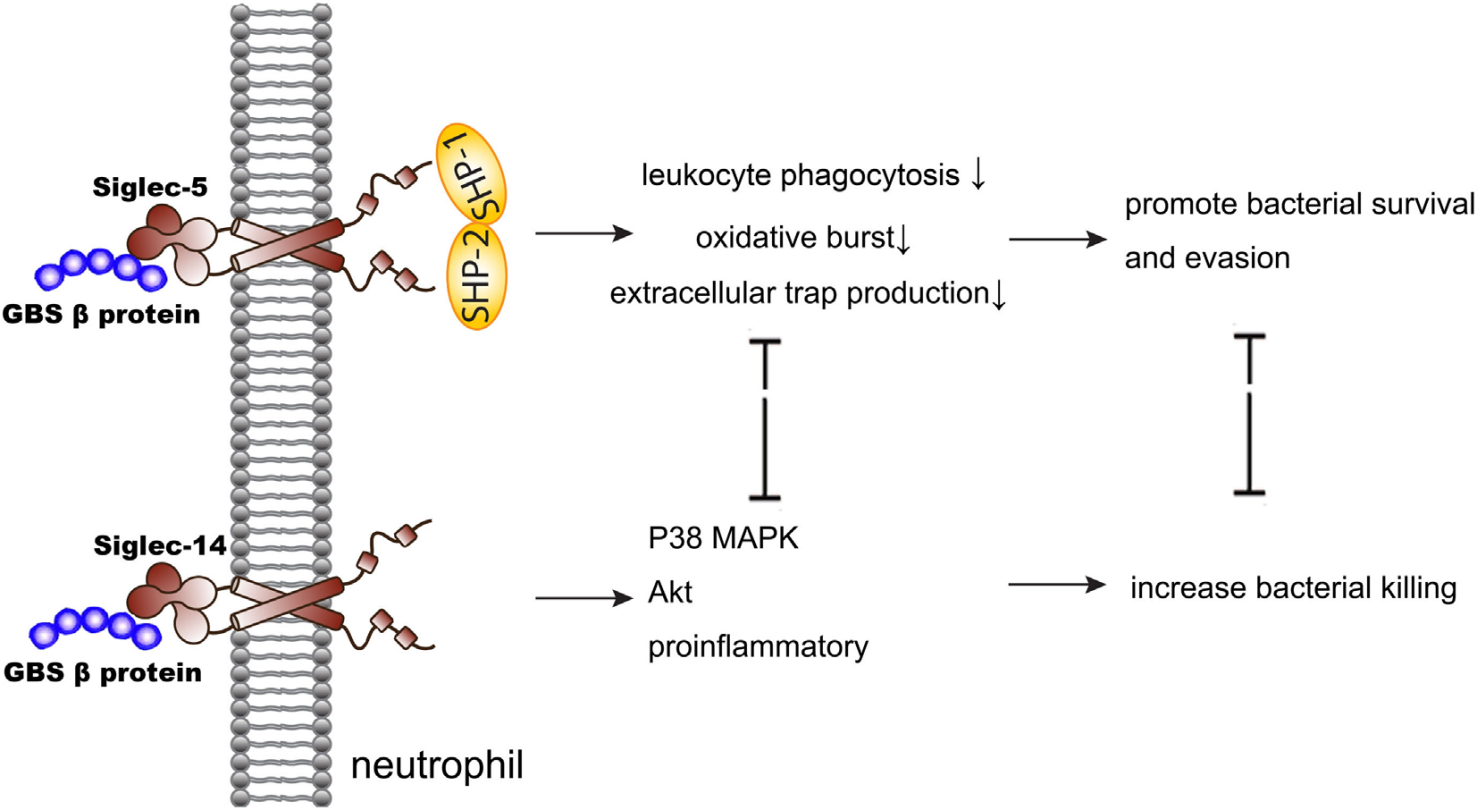
Siglec-5 and Siglec-14 serve as a paired receptor counteracting each other in regulating immune response of GBS infection. GBS, group B *Streptococcus*; MAPK, mitogen-activated protein kinase.

Siglec-5 and Siglec-14, a paired receptor system in the Siglecs family expressed on monocytes and neutrophils, share almost identical ligand-binding domains but have opposing effects in the regulation of host immunity. This idea was discovered in the research of group B *Streptococcus* (GBS) infection (Figure 1). Early study showed that GBS β protein bound to Siglec-5 and promoted bacterial survival through impairing human leukocyte phagocytosis, oxidative burst, and extracellular trap production (31). Five years later, Ali et al. discovered that Siglec-14 also involved in the GBS infection as a paired receptor with Siglec-5. β protein of GBS bound to both Siglec-5 and Siglec-14 on neutrophils, and Siglec-14 counteracted pathogen-induced host immune suppression by activating p38 mitogen-activated protein kinase (MAPK) and Akt signaling pathways (32). As Siglec-14 is not expressed by all people, homozygous Siglec-14-null neutrophils are more susceptible to GBS immune subversion (32). This idea was also confirmed in the research of chronic obstructive pulmonary disease (COPD). Loss of Siglec-14 reduces the risk of COPD exacerbation (33), and inhaled corticosteroids could exert two opposite effects depending on the patients’ phenotypes of Siglec-5 and Siglec-14 (34). What’s more, a recently study found that heat shock protein (Hsp) 70, a danger-associated molecular pattern (DAMP), could bind to both Siglec-5 and Siglec-14 and play a two-way role in the immune modulation (35). This may explain the contradictory conclusions on the function of extracellular Hsp70 in inflammation (36–38). In brief, the bidirectional action played by Siglec-5 and Siglec-14 involved both neutrophils function and Hsp70 modulation in infection.

## Siglec-7

Siglec-7 (CD328) is constitutively expressed on natural killer (NK) cells, mast cells, basophils, and platelets. It has been proven as a very important regulator of the immune response through inhibiting NK cells activation, regulating apoptosis and death, and affecting IgE-mediated mast cells and basophils activation (39–41). In sepsis, Siglec-7 acts as a target of suppressor of cytokine signaling 3 (SOCS3) and amplify inflammation through activating monocytes (42). SOCS3 in the spleen, lung, and peritoneal leukocytes is up-regulated during sepsis (43). SOCS3 binds the phosphorylated ITIMs carried by Siglec-7 and blocks Siglec-7 mediated inhibition of cytokine-induced proliferation. This also contributes to the exaggerated inflammatory response induced by pro-inflammatory cytokines during infection (42).

Some pathogens escape host immune response through binding to Siglec-7 with sialic acids expressed on their surface. Varchetta et al. demonstrated that Siglec-7 activated a monocyte-mediated inflammatory and produced high level of pro-inflammatory cytokines and chemokines through phosphorylation of the extracellular signal-regulated kinase (ERK) pathway following pathogen recognition. What’s more, Siglec-7 also participated in generating a monocyte-mediated inflammatory when encountering pathogens not expressing sialylated glycans. This phenomenon may provide an alternative mechanism that Siglec-7 involved in sepsis (44).

## Siglec-9

**Figure 2:**
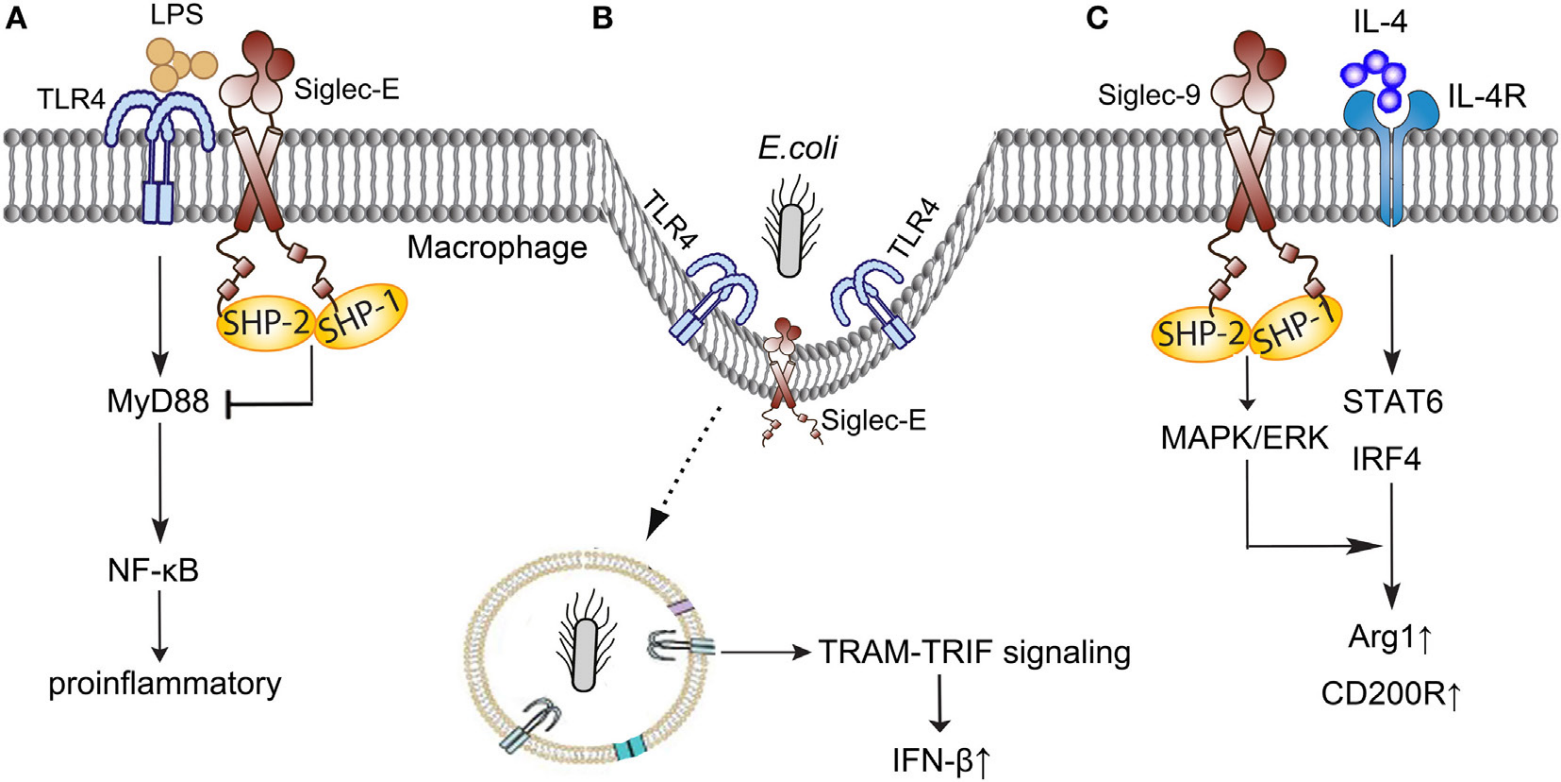
Siglec-E/9 in the immune regulation of sepsis. **(A)** Siglec-E negatively regulates TLR4 responses in a MyD88-specific manner following LPS stimulation. **(B)** Siglec-E provides immune balance in inflammation when participating in the Escherichia *coli*-induced endocytosis of TLR4. **(C)** Siglec-9 enhances IL-4-induced Arg-1 and CD200R production through MAPK/ERK pathways. TLR, toll-like receptor; LPS, lipopolysaccharides; MyD, myeloid differentiation factor; NF-κB, nuclear factor-kappa B; TRIF, TIR-domain-containing adapter-inducing interferon-β; TRAM, TRIF-related adaptor molecule; IFN-β, interferon-β; IL-4, interleukin 4; MAPK/ERK, mitogen-activated protein kinase/extracellular signal-regulated kinase; STAT, signal transducer and activator of transcription; IRF, interferon regulatory factor; Arg-1, arginase1.

Siglec-9, Siglec-E in murine, the major CD33-related Siglec, is mainly expressed on neutrophils, monocytes, macrophages, and dendritic cells (45), and involves in the pathogenesis of sepsis through interacting with TLR4, regulating the polarization of macrophages, and inhibiting the stimulation of neutrophils (Figure 2).

Broad and direct interaction exist between TLR4 and Siglec-E (46). Murine Siglec-E is induced by TLR4 in a myeloid differentiation factor (MyD) 88-specific manner and negatively regulates TLR4 responses following lipopolysaccharides (LPS) stimulation (47). A recent study discovered that Siglec-E participated in the Escherichia *coli*-induced endocytosis of TLR4, and provided an immune balance in inflammation (48). Siglec-E deficient dendritic cells failed to internalize the TLR4 and resulted in high levels of pro-inflammatory cytokines through nuclear factor-kappa B (NF-κB) and MAPK p38 signal pathway when infected with *E. coli* (48). Taken together, Siglec-E plays a novel role in controlling the septic response with TLR4 and helps to maintain a healthy cytokine balance following infection.

Macrophages polarization plays a pivotal role in the pathogenesis of sepsis, and regulating the phenotypes of macrophages in the different stages of sepsis holds a great potential in the treatment of sepsis (49, 50). Recent studies shown that Siglec-9 enhanced induction of Arg-1 through MAPK/ERK pathways in the stimulation of interleukin 4 (IL-4) (51). Siglec-9 enhanced IL-4-induced CD200R expression and inhibited LPS-induced CCR7 in human macrophages (52). However, the detailed mechanisms under Siglec-E and macrophages polarization in sepsis need to be further elucidated.

As an important regulator expressed on neutrophils, Siglec-E function as an inhibitory receptor on the neutrophils stimulated by LPS. McMillan et al. (53) demonstrated that Siglec-E inhibited the β2-integrin-dependent neutrophil recruitment to the lung and enhanced nicotinamide adenine dinucleotide phosphate-oxidase (NADPH) oxidase activation and reactive oxygen species production *via* Akt activation following exposure to LPS. What is interesting, the reason of neutrophils become much easier activated after separation from whole blood also involved in Siglec-9. A recent study discovered that the abundant sialoglycoprotein on erythrocytes engaged neutrophil Siglec-9 and dampened the innate immune cell activation (54).

What’s more, a recent study using mouse and Chinese hamster ovary cells discovered a new role for Siglec-E/Siglec-9 (55). Siglec-E/Siglec-9 could specifically bind to vascular adhesion protein-1 (VAP-1), an endothelial cell molecule involved in granulocyte migration to sites of inflammation. Using ^68^Gallium-labeled peptide of Siglec-9 to detect VAP-1 in vasculature as an imaging tool in inflammation in positron emission tomography will give great help in the treatment of inflammatory diseases.

Recently, great progress has been made in Siglec-E targeting therapy of sepsis. Spence et al. created nanoparticles decorated with sialic acid and developed a novel strategy to control inflammation. From human monocytes and macrophages *in vitro* model and human *ex vivo* model of lung injury, they revealed that those special nanoparticles blocked the production of inflammatory mediators induced by LPS in a Siglec-E-dependent manner through enhancing the oligomerization of Siglec-E receptor on macrophages (56). Another study from human peripheral blood mononuclear cell-derived macrophages showed that a human anti-Siglec-9 Fab fragment named hS9-Fab03, specially bound to Siglec-9 antigen with high affinity and attenuated LPS-induced pro-inflammatory cytokines production (57). Those discovery confirmed that Siglec-E/Siglec-9 as a druggable anti-inflammatory therapeutic target for sepsis.

## Siglec-10

**Figure 3:**
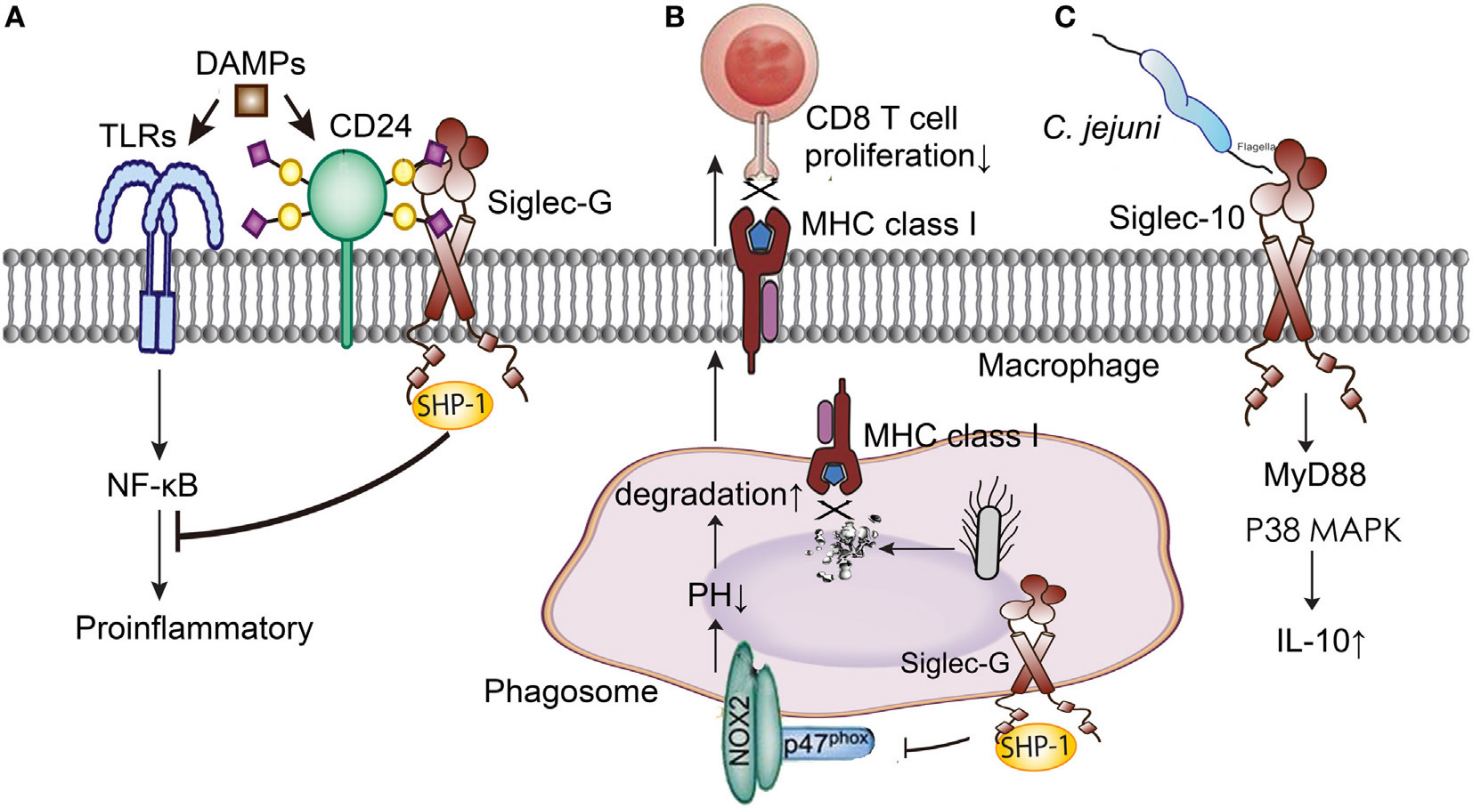
Siglec-G/10 in the immune regulation of sepsis. **(A)** Siglec-G decreases the inflammation induced by DAMPs through NF-κB signal pathway with the help of CD24. **(B)** Siglec-G inhibits CD 8T cells proliferation through impairing MHC class I-peptide complexes formation. **(C)** Siglec-10 increases IL-10 production through MyD88 and p38 MAPK signaling pathways in Campylobacter *jejuni* infection. DAMPs, danger-associated molecular patterns; TLRs, toll-like receptors; NF-κB, nuclear factor-kappa B; MHC, major histocompatibility complex; MyD, myeloid differentiation factor; MAPK, mitogen-activated protein kinase; IL-10, interleukin 10.

Siglec-10, Siglec-G in murine, is broadly expressed on B cells, dendritic cells, and macrophages subsets, which is also a member of the CD33-related Siglecs family (58). It involves in the process of innate and adaptive immune response, and plays an anti-inflammatory role in sepsis through increasing IL-10 expression, interacting with CD24, inhibiting dendritic cell cross presentation, and weakening B cell signaling (Figure 3).

Siglec-10 involved in the *Campylobacter jejuni* infection and promoted an anti-inflammatory function through binding to *C. jejuni* and purified flagellum and increasing IL-10 expression by MyD88 and p38 MAPK signaling pathways (59). Siglec-G also mediated an immune evasion pathway in RNA virus infection. Chen et al. discovered that RNA virus specifically up-regulated Siglec-G expression in macrophages by RIG-I or NF-κB-dependent mechanisms. Siglec-G recruited SHP-2 and E3 ubiquitin ligase c-Cbl to RIG-I and induced RIG-I degradation *via* K48-linked ubiquitination at Lys813 by c-Cbl. The increased Siglec-G led to the persistence of RNA virus infection and severe immunopathology through the suppression of IFN-I production (60).

CD24 protects the host against the exaggerated inflammatory response in sepsis (61). CD24 is a small glycosyl-phosphoinositol-anchored protein that is able to provide costimulatory signals to T cells. In sepsis, CD24 associates with DAMPs, such as high-mobility group box 1, Hsp70, and Hsp90, negatively regulates their stimulatory activity and inhibits NF-κB activation through association with Siglec-G (62). What’s more, microbial sialidase targeting Siglec-G blocks the CD24-Siglec-G pathway and exacerbates inflammation. Using sialidase inhibitors to prevent disrupting sialic acid-based pattern recognition protected mice against cecal ligation and puncture (CLP) induced sepsis, and this process depended on the CD24 and Siglec-G interaction (63, 64). The pathogenesis of sepsis involves multiple inflammatory mediators and a lot of them are regulated by the interaction of CD24 and Siglec-G. Therefore, sialidase inhibitors targeting CD24-Siglec-G interaction has a great clinical potential in the treatment of sepsis.

Siglec-G expressed on dendritic cells also contributed to the initiation of antigen response by T cells. Siglec-G inhibits cross-presenting extracellular antigens with CD8 T cells by impairing major histocompatibility complex class I-peptide complexes formation. This process involves recruiting the phosphatase SHP-1 by Siglec-G, dephosphorylating the NADPH oxidase component p47phox, and inhibiting the activation of NOX2 on phagosomes (65). Soluble CD52 released by phospholipase C bound to Siglec-10 and impaired phosphorylation of the T cell receptor associated kinases Lck and Zap70 and T cell activation, which was distinct from regular T cells (66).

Siglec-G is also broadly expressed on B cells, and plays as a negative regulator of B cell receptor (BCR)-mediated signaling in inflammation. Siglec-G binds to the BCR on the B cell surface *via* interaction with sialic acid ligands, and controls B cell tolerance (67). Siglec-G^−/−^ B-1a cells display an altered BCR repertoire and a higher expression levels of the transcription factor, and show a lower level of spontaneous apoptosis and a prolonged life span (68). Hence, Siglec-G negatively regulates the inflammation through decreasing B-1a cell population, weakening B-1 cell signaling, and shifting the immunoglobulin repertoire secreted by B-1 cells.

## Summary

Immune disorder contributes to the different stages of sepsis, while the Siglecs play significant roles in the immune regulation. There are heavy conjugations between Siglecs and the pathogenesis and therapy of sepsis. Siglec-1, Siglec-5, and Siglec-14 play bidirectional roles in sepsis through modulation of inflammation and immunity. Siglec-2 involves in B cell and T cell response during infection and regulates the immune balance. Siglec-9 helps endocytosis of TLR4, regulates macrophages polarization, and inhibits the function of neutrophils during infection. Siglec-10 inhibits DAMPs-induced inflammation, helps the initiation of antigen response by T cells, and decreases B-1a cell population to weaken inflammation.

However, our current knowledge of Siglecs in the pathogenesis and therapy of sepsis is in its infancy. Most research has focused on the pathogens-related sepsis, but the researches using CLP model, the golden standard of sepsis, are few and far between. Therefore, more Siglecs-related studies using CLP model are in urgent demand. In addition, more researches are also needed in the function of T cells and NK cells with the participation of Siglecs in sepsis. As the cytokines storms stage and the immunosuppression stage of sepsis are totally different immune state, investigating the different functions of Siglecs in the different stages of sepsis is also very meaningful. Collectively, investigating the roles played by Siglecs in the immune response will not only contribute to the therapy of sepsis, but also hold great potentials in the treatment of other inflammatory diseases.

## Author Contributions

Y-CL and M-MY drafted the manuscript and performed a literature review. S-TS and Y-FC were served as chief physicians. All authors read and approved the final manuscript.

## Conflict of Interest Statement

The authors declare that the research was conducted in the absence of any commercial or financial relationships that could be construed as a potential conflict of interest.

## Abbreviations

Arg-1, arginase1; BCR, B cell receptor; CCR7, chemokine receptor 7; CLP, cecal ligation and puncture; COPD, chronic obstructive pulmonary disease; DAMPs, danger-associated molecular patterns; dLNs, draining lymph nodes; ERK, extracellular signal-regulated kinase; GBS, group B *Streptococcus*; HIV, human immunodeficiency virus; HMGB1, high-mobility group box 1; Hsp, heat shock protein; IFN, interferon; IL, interleukin; IRF, interferon regulatory factor; ITIMs, immunoreceptor tyrosine-based inhibitory motifs; LCMV, lymphocytic choriomeningitis virus; LPS, lipopolysaccharides; MAPK, mitogen-activated protein kinase; MAPK/ERK, mitogen-activated protein kinase/extracellular signal-regulated kinase; MHC, major histocompatibility complex; MLV, murine leukemia viruses; MODS, multiple organ dysfunction syndrome; MyD, myeloid differentiation factor; NF-κB, nuclear factor-kappa B; PD-L1, programmed death-ligand 1; PET, positron emission tomography; PSGL1, P-selectin glycoprotein ligand-1; ROS, reactive oxygen species; Siglecs, sialic acid-binding immunoglobulin-type lectins; SOCS3, suppressor of cytokine signaling; STAT, signal transducer and activator of transcription; TGF-β, transforming growth factor-β; TLR, toll-like receptor; TRAM, TRIF-related adaptor molecule; IFN-β, interferon-β; TRIF, TIR-domain-containing adapter-inducing interferon-β; VAP-1, vascular adhesion protein-1; VCCs, virus-containing compartments; WNV, West Nile virus.

## References

[1] Schauer R Sialic acids as regulators of molecular and cellular interactions. Curr Opin Struct Biol (2009) 19(5):507–14. 10.1016/j.sbi.2009.06.003 19699080 PMC7127376

[2] Macauley MS Crocker PR Paulson JC. Siglec-mediated regulation of immune cell function in disease. Nat Rev Immunol (2014) 14(10):653–66. 10.1038/nri3737 25234143 PMC4191907

[3] Pearce OM Laubli H. Sialic acids in cancer biology and immunity. Glycobiology (2016) 26(2):111–28. 10.1093/glycob/cwv097 26518624

[4] Mahajan VS Pillai S. Sialic acids and autoimmune disease. Immunol Rev (2016) 269(1):145–61. 10.1111/imr.12344 26683151 PMC4769436

[5] Chang YC Nizet V. The interplay between Siglecs and sialylated pathogens. Glycobiology (2014) 24(9):818–25. 10.1093/glycob/cwu067 24996821 PMC4168292

[6] Cao H de Bono B Belov K Wong ES Trowsdale J Barrow AD. Comparative genomics indicates the mammalian CD33rSiglec locus evolved by an ancient large-scale inverse duplication and suggests all Siglecs share a common ancestral region. Immunogenetics (2009) 61(5):401–17. 10.1007/s00251-009-0372-0 19337729

[7] Rhodes A Evans LE Alhazzani W Levy MM Antonelli M Ferrer R Surviving sepsis campaign: international guidelines for management of sepsis and septic shock: 2016. Crit Care Med (2017) 45(3):486–552. 10.1097/CCM.0000000000002255 28098591

[8] Lagu T Rothberg MB Shieh MS Pekow PS Steingrub JS Lindenauer PK. Hospitalizations, costs, and outcomes of severe sepsis in the United States 2003 to 2007. Crit Care Med (2012) 40(3):754–61. 10.1097/CCM.0b013e318232db65 21963582

[9] Singer M Deutschman CS Seymour CW Shankar-Hari M Annane D Bauer M The third international consensus definitions for sepsis and septic shock (sepsis-3). JAMA (2016) 315(8):801–10. 10.1001/jama.2016.0287 26903338 PMC4968574

[10] Shankar-Hari M Phillips GS Levy ML Seymour CW Liu VX Deutschman CS Developing a new definition and assessing new clinical criteria for septic shock: for the third international consensus definitions for sepsis and septic shock (sepsis-3). JAMA (2016) 315(8):775–87. 10.1001/jama.2016.0289 26903336 PMC4910392

[11] Seymour CW Liu VX Iwashyna TJ Brunkhorst FM Rea TD Scherag A Assessment of clinical criteria for sepsis: for the third international consensus definitions for sepsis and septic shock (sepsis-3). JAMA (2016) 315(8):762–74. 10.1001/jama.2016.0288 26903335 PMC5433435

[12] Martin GS Mannino DM Eaton S Moss M. The epidemiology of sepsis in the United States from 1979 through 2000. N Engl J Med (2003) 348(16):1546–54. 10.1056/NEJMoa022139 12700374

[13] Angus DC Linde-Zwirble WT Lidicker J Clermont G Carcillo J Pinsky MR. Epidemiology of severe sepsis in the United States: analysis of incidence, outcome, and associated costs of care. Crit Care Med (2001) 29(7):1303–10. 10.1097/00003246-200107000-00002 11445675

[14] Boomer JS To K Chang KC Takasu O Osborne DF Walton AH Immunosuppression in patients who die of sepsis and multiple organ failure. JAMA (2011) 306(23):2594–605. 10.1001/jama.2011.1829 22187279 PMC3361243

[15] Deutschman CS Tracey KJ. Sepsis: current dogma and new perspectives. Immunity (2014) 40(4):463–75. 10.1016/j.immuni.2014.04.001 24745331

[16] Hotchkiss RS Monneret G Payen D. Sepsis-induced immunosuppression: from cellular dysfunctions to immunotherapy. Nat Rev Immunol (2013) 13(12):862–74. 10.1038/nri3552 24232462 PMC4077177

[17] Izquierdo-Useros N Lorizate M Contreras FX Rodriguez-Plata MT Glass B Erkizia I Sialyllactose in viral membrane gangliosides is a novel molecular recognition pattern for mature dendritic cell capture of HIV-1. PLoS Biol (2012) 10(4):e1001315. 10.1371/journal.pbio.1001315 22545022 PMC3335875

[18] Gummuluru S Pina Ramirez NG Akiyama H. CD169-dependent cell-associated HIV-1 transmission: a driver of virus dissemination. J Infect Dis (2014) 210(Suppl 3):S641–7. 10.1093/infdis/jiu442 25414418 PMC4303078

[19] Gupta P Lai SM Sheng J Tetlak P Balachander A Claser C Tissue-resident CD169(+) macrophages form a crucial front line against plasmodium infection. Cell Rep (2016) 16(6):1749–61. 10.1016/j.celrep.2016.07.010 27477286

[20] Shaabani N Duhan V Khairnar V Gassa A Ferrer-Tur R Haussinger D CD169+ macrophages regulate PD-L1 expression via type I interferon and thereby prevent severe immunopathology after LCMV infection. Cell Death Dis (2016) 7(11):e2446. 10.1038/cddis.2016.350 27809306 PMC5260878

[21] Wu Y Lan C Ren D Chen GY Induction of Siglec-1 by endotoxin tolerance suppresses the innate immune response by promoting TGF-beta1 production. J Biol Chem (2016) 291(23):12370–82. 10.1074/jbc.M116.721258 27129263 PMC4933283

[22] Sewald X Ladinsky MS Uchil PD Beloor J Pi R Herrmann C Retroviruses use CD169-mediated trans-infection of permissive lymphocytes to establish infection. Science (2015) 350(6260):563–7. 10.1126/science.aab2749 26429886 PMC4651917

[23] Akiyama H Ramirez NG Gudheti MV Gummuluru S. CD169-mediated trafficking of HIV to plasma membrane invaginations in dendritic cells attenuates efficacy of anti-gp120 broadly neutralizing antibodies. PLoS Pathog (2015) 11(3):e1004751. 10.1371/journal.ppat.1004751 25760631 PMC4356592

[24] Walker JA Smith KG. CD22: an inhibitory enigma. Immunology (2008) 123(3):314–25. 10.1111/j.1365-2567.2007.02752.x 18067554 PMC2433339

[25] Gjertsson I Nitschke L Tarkowski A. The role of B cell CD22 expression in *Staphylococcus aureus* arthritis and sepsis. Microbes Infect (2004) 6(4):377–82. 10.1016/j.micinf.2003.12.013 15050965

[26] Jiang YN Cai X Zhou HM Jin WD Zhang M Zhang Y Diagnostic and prognostic roles of soluble CD22 in patients with Gram-negative bacterial sepsis. Hepatobiliary Pancreat Dis Int (2015) 14(5):523–9. 10.1016/S1499-3872(15)60394-0 26459729

[27] Jiang Y Zhou H Ma D Chen ZK Cai X. MicroRNA-19a and CD22 comprise a feedback loop for B cell response in sepsis. Med Sci Monit (2015) 21:1548–55. 10.12659/MSM.894321 26017478 PMC4459571

[28] Ma DY Suthar MS Kasahara S Gale M Jr Clark EA. CD22 is required for protection against West Nile virus infection. J Virol (2013) 87(6):3361–75. 10.1128/JVI.02368-12 23302871 PMC3592166

[29] Nitschke L Suppressing the antibody response with Siglec ligands. N Engl J Med (2013) 369(14):1373–4. 10.1056/NEJMcibr1308953 24088100

[30] Kreitman RJ Squires DR Stetler-Stevenson M Noel P FitzGerald DJ Wilson WH Phase I trial of recombinant immunotoxin RFB4(dsFv)-PE38 (BL22) in patients with B-cell malignancies. J Clin Oncol (2005) 23(27):6719–29. 10.1200/JCO.2005.11.437 16061911

[31] Carlin AF Chang YC Areschoug T Lindahl G Hurtado-Ziola N King CC Group B *Streptococcus* suppression of phagocyte functions by protein-mediated engagement of human Siglec-5. J Exp Med (2009) 206(8):1691–9. 10.1084/jem.20090691 19596804 PMC2722167

[32] Ali SR Fong JJ Carlin AF Busch TD Linden R Angata T Siglec-5 and Siglec-14 are polymorphic paired receptors that modulate neutrophil and amnion signaling responses to group B *Streptococcus*. J Exp Med (2014) 211(6):1231–42. 10.1084/jem.20131853 24799499 PMC4042635

[33] Angata T Ishii T Motegi T Oka R Taylor RE Soto PC Loss of Siglec-14 reduces the risk of chronic obstructive pulmonary disease exacerbation. Cell Mol Life Sci (2013) 70(17):3199–210. 10.1007/s00018-013-1311-7 23519826 PMC3718857

[34] Wielgat P Mroz RM Stasiak-Barmuta A Szepiel P Chyczewska E Braszko JJ Inhaled corticosteroids increase Siglec-5/14 expression in sputum cells of COPD patients. Adv Exp Med Biol (2015) 839:1–5. 10.1007/5584_2014_51 25252903

[35] Fong JJ Sreedhara K Deng L Varki NM Angata T Liu Q Immunomodulatory activity of extracellular Hsp70 mediated via paired receptors Siglec-5 and Siglec-14. EMBO J (2015) 34(22):2775–88. 10.15252/embj.201591407 26459514 PMC4682649

[36] Gao B Tsan MF. Induction of cytokines by heat shock proteins and endotoxin in murine macrophages. Biochem Biophys Res Commun (2004) 317(4):1149–54. 10.1016/j.bbrc.2004.03.160 15094389

[37] Gao B Tsan MF. Endotoxin contamination in recombinant human heat shock protein 70 (Hsp70) preparation is responsible for the induction of tumor necrosis factor alpha release by murine macrophages. J Biol Chem (2003) 278(1):174–9. 10.1074/jbc.M208742200 12403778

[38] Asea A Kraeft SK Kurt-Jones EA Stevenson MA Chen LB Finberg RW HSP70 stimulates cytokine production through a CD14-dependant pathway, demonstrating its dual role as a chaperone and cytokine. Nat Med (2000) 6(4):435–42. 10.1038/74697 10742151

[39] Shao JY Yin WW Zhang QF Liu Q Peng ML Hu HD Siglec-7 defines a highly functional natural killer cell subset and inhibits cell-mediated activities. Scand J Immunol (2016) 84(3):182–90. 10.1111/sji.12455 27312286

[40] Mizrahi S Gibbs BF Karra L Ben-Zimra M Levi-Schaffer F Siglec-7 is an inhibitory receptor on human mast cells and basophils. J Allergy Clin Immunol (2014) 134(1):230–3. 10.1016/j.jaci.2014.03.031 24810846

[41] Nguyen KA Hamzeh-Cognasse H Palle S Anselme-Bertrand I Arthaud CA Chavarin P Role of Siglec-7 in apoptosis in human platelets. PLoS One (2014) 9(9):e106239. 10.1371/journal.pone.0106239 25230315 PMC4167548

[42] Orr SJ Morgan NM Buick RJ Boyd CR Elliott J Burrows JF SOCS3 targets Siglec 7 for proteasomal degradation and blocks Siglec 7-mediated responses. J Biol Chem (2007) 282(6):3418–22. 10.1074/jbc.C600216200 17138568

[43] Grutkoski PS Chen Y Chung CS Ayala A. Sepsis-induced SOCS-3 expression is immunologically restricted to phagocytes. J Leukoc Biol (2003) 74(5):916–22. 10.1189/jlb.0303108 12960286 PMC2254146

[44] Varchetta S Brunetta E Roberto A Mikulak J Hudspeth KL Mondelli MU Engagement of Siglec-7 receptor induces a pro-inflammatory response selectively in monocytes. PLoS One (2012) 7(9):e45821. 10.1371/journal.pone.0045821 23029261 PMC3461047

[45] Siddiqui S Schwarz F Springer S Khedri Z Yu H Deng L Studies on the detection, expression, glycosylation, dimerization, and ligand binding properties of mouse Siglec-E. J Biol Chem (2017) 292(3):1029–37. 10.1074/jbc.M116.738351 27920204 PMC5247637

[46] Chen GY Brown NK Wu W Khedri Z Yu H Chen X Broad and direct interaction between TLR and Siglec families of pattern recognition receptors and its regulation by Neu1. Elife (2014) 3:e04066. 10.7554/eLife.04066 25187624 PMC4168287

[47] Boyd CR Orr SJ Spence S Burrows JF Elliott J Carroll HP Siglec-E is up-regulated and phosphorylated following lipopolysaccharide stimulation in order to limit TLR-driven cytokine production. J Immunol (2009) 183(12):7703–9. 10.4049/jimmunol.0902780 19933851 PMC5580793

[48] Wu Y Ren D Chen GY. Siglec-E negatively regulates the activation of TLR4 by controlling its endocytosis. J Immunol (2016) 197(8):3336–47. 10.4049/jimmunol.1600772 27619995 PMC5101162

[49] Liu YC Zou XB Chai YF Yao YM. Macrophage polarization in inflammatory diseases. Int J Biol Sci (2014) 10(5):520–9. 10.7150/ijbs.8879 24910531 PMC4046879

[50] Liu YC Yao FH Chai YF Dong N Sheng ZY Yao YM. Xuebijing injection promotes M2 polarization of macrophages and improves survival rate in septic mice. Evid Based Complement Alternat Med (2015) 2015:352642. 10.1155/2015/352642 26064161 PMC4441998

[51] Higuchi H Shoji T Murase Y Iijima S Nishijima K. Siglec-9 modulated IL-4 responses in the macrophage cell line RAW264. Biosci Biotechnol Biochem (2016) 80(3):501–9. 10.1080/09168451.2015.1104238 26540411

[52] Higuchi H Shoji T Iijima S Nishijima K. Constitutively expressed Siglec-9 inhibits LPS-induced CCR7, but enhances IL-4-induced CD200R expression in human macrophages. Biosci Biotechnol Biochem (2016) 80(6):1141–8. 10.1080/09168451.2016.1146070 26923638

[53] McMillan SJ Sharma RS Richards HE Hegde V Crocker PR Siglec-E promotes beta2-integrin-dependent NADPH oxidase activation to suppress neutrophil recruitment to the lung. J Biol Chem (2014) 289(29):20370–6. 10.1074/jbc.M114.574624 24895121 PMC4106349

[54] Lizcano A Secundino I Dohrmann S Corriden R Rohena C Diaz S Erythrocyte sialoglycoproteins engage Siglec-9 on neutrophils to suppress activation. Blood (2017) 129(23):3100–10. 10.1182/blood-2016-11-751636 28416510 PMC5465837

[55] Aalto K Autio A Kiss EA Elima K Nymalm Y Veres TZ Siglec-9 is a novel leukocyte ligand for vascular adhesion protein-1 and can be used in PET imaging of inflammation and cancer. Blood (2011) 118(13):3725–33. 10.1182/blood-2010-09-311076 21821708 PMC3833035

[56] Spence S Greene MK Fay F Hams E Saunders SP Hamid U Targeting Siglecs with a sialic acid-decorated nanoparticle abrogates inflammation. Sci Transl Med (2015) 7(303):303ra140. 10.1126/scitranslmed.aab3459 26333936

[57] Chu S Zhu X You N Zhang W Zheng F Cai B The Fab fragment of a human anti-Siglec-9 monoclonal antibody suppresses lps-induced inflammatory responses in human macrophages. Front Immunol (2016) 7:649. 10.3389/fimmu.2016.00649 28082984 PMC5183739

[58] Chen GY Brown NK Zheng P Liu Y. Siglec-G/10 in self-nonself discrimination of innate and adaptive immunity. Glycobiology (2014) 24(9):800–6. 10.1093/glycob/cwu068 24996822 PMC4116048

[59] Stephenson HN Mills DC Jones H Milioris E Copland A Dorrell N Pseudaminic acid on *Campylobacter jejuni* flagella modulates dendritic cell IL-10 expression via Siglec-10 receptor: a novel flagellin-host interaction. J Infect Dis (2014) 210(9):1487–98. 10.1093/infdis/jiu287 24823621 PMC4195440

[60] Chen W Han C Xie B Hu X Yu Q Shi L Induction of Siglec-G by RNA viruses inhibits the innate immune response by promoting RIG-I degradation. Cell (2013) 152(3):467–78. 10.1016/j.cell.2013.01.011 23374343

[61] Parlato M Souza-Fonseca-Guimaraes F Philippart F Misset B Captain Study G Adib-Conquy M CD24-triggered caspase-dependent apoptosis via mitochondrial membrane depolarization and reactive oxygen species production of human neutrophils is impaired in sepsis. J Immunol (2014) 192(5):2449–59. 10.4049/jimmunol.1301055 24501201

[62] Chen GY Tang J Zheng P Liu Y. CD24 and Siglec-10 selectively repress tissue damage-induced immune responses. Science (2009) 323(5922):1722–5. 10.1126/science.1168988 19264983 PMC2765686

[63] Chen GY Chen X King S Cavassani KA Cheng J Zheng X Amelioration of sepsis by inhibiting sialidase-mediated disruption of the CD24-SiglecG interaction. Nat Biotechnol (2011) 29(5):428–35. 10.1038/nbt.1846 21478876 PMC4090080

[64] Paulson JC Kawasaki N Sialidase inhibitors DAMPen sepsis. Nat Biotechnol (2011) 29(5):406–7. 10.1038/nbt.1859 21552240 PMC3565534

[65] Ding Y Guo Z Liu Y Li X Zhang Q Xu X The lectin Siglec-G inhibits dendritic cell cross-presentation by impairing MHC class I-peptide complex formation. Nat Immunol (2016) 17(10):1167–75. 10.1038/ni.3535 27548433

[66] Bandala-Sanchez E Zhang Y Reinwald S Dromey JA Lee BH Qian J T cell regulation mediated by interaction of soluble CD52 with the inhibitory receptor Siglec-10. Nat Immunol (2013) 14(7):741–8. 10.1038/ni.2610 23685786

[67] Nitschke L. Siglec-G is a B-1 cell inhibitory receptor and also controls B cell tolerance. Ann N Y Acad Sci (2015) 1362:117–21. 10.1111/nyas.12826 26194636

[68] Jellusova J Duber S Guckel E Binder CJ Weiss S Voll R Siglec-G regulates B1 cell survival and selection. J Immunol (2010) 185(6):3277–84. 10.4049/jimmunol.1001792 20729333

